# Histamine-HisCl1 Receptor Axis Regulates Wake-Promoting Signals in *Drosophila melanogaster*

**DOI:** 10.1371/journal.pone.0068269

**Published:** 2013-07-03

**Authors:** Yangkyun Oh, Donghoon Jang, Jun Young Sonn, Joonho Choe

**Affiliations:** Department of Biological Sciences, Korea Advanced Institute of Science and Technology, Daejeon, Republic of Korea; Wake Forest University, United States of America

## Abstract

Histamine and its two receptors, histamine-gated chloride channel subunit 1 (HisCl1) and ora transientless (Ort), are known to control photoreception and temperature sensing in *Drosophila*. However, histamine signaling in the context of neural circuitry for sleep-wake behaviors has not yet been examined in detail. Here, we obtained mutant flies with compromised or enhanced histamine signaling and tested their baseline sleep. Hypomorphic mutations in histidine decarboxylase (HDC), an enzyme catalyzing the conversion from histidine to histamine, caused an increase in sleep duration. Interestingly, *hisCl1* mutants but not *ort* mutants showed long-sleep phenotypes similar to those in *hdc* mutants. Increased sleep duration in *hisCl1* mutants was rescued by overexpressing *hisCl1* in circadian pacemaker neurons expressing a neuropeptide pigment dispersing factor (PDF). Consistently, RNA interference (RNAi)-mediated depletion of *hisCl1* in PDF neurons was sufficient to mimic *hisCl1* mutant phenotypes, suggesting that PDF neurons are crucial for sleep regulation by the histamine-HisCl1 signaling. Finally, either *hisCl1* mutation or genetic ablation of PDF neurons dampened wake-promoting effects of elevated histamine signaling via direct histamine administration. Taken together, these data clearly demonstrate that the histamine-HisCl1 receptor axis can activate and maintain the wake state in *Drosophila* and that wake-activating signals may travel via the PDF neurons.

## Introduction

Although sleep is known to be crucial for the physiology and life of an animal [1], the precise regulatory mechanisms that govern sleep are not yet fully understood. Sleep is regulated by the circadian rhythm and the homeostatic systems, which control the timing and need for sleep [2]. In mammals, hypothalamic neurons constitute one of the major control centers of sleep and wakefulness; in the hypothalamus, wake-promoting neurons and sleep-promoting neurons create a feedback loop that modulates sleep and wakefulness [3]–[7].

In mammals, histamine, a monoamine synthesized from histidine by histidine decarboxylase (HDC), is a major neurotransmitter that regulates learning, immune reactions [8], and sleep-wake behavior [9], [10]. Histaminergic neurons control the wakefulness in the hypothalamus, especially in the tuberomammillary nucleus (TMN). There are four mammalian histamine receptors belonging to the family of rhodopsin-like G-protein-coupled receptors [11]: H_1_, H_2_, H_3_ and H_4_. The H_1_ receptor, which is coupled to the phospholipase C pathway for the activation of calcium signals [12], is known to play an important role in cognitive function and activation of the waking state [13], [14]. H_1_ receptor antagonists have been used to treat allergic symptoms, but often show drowsiness as a common side effect; some (e.g., doxylamine succinate and diphenhydramine) are even used to treat insomnia. The H_2_ receptor, which activates cAMP signaling by activating adenylate cyclase, has memory modulating effects, but has little impact on sleep/wake regulation [13], [15]. The H_3_ receptor acts as an auto-receptor in presynaptic histaminergic neurons and controls histamine turnover through feedback inhibition of histamine synthesis and release. The H_3_ receptor is located on presynaptic terminals and can affect the sleep/wake cycle as well as learning and memory by controlling histamine synthesis and release [16]–[18]. Finally, the H_4_ receptor is highly expressed in bone marrow and white blood cells and mediates several immune responses [19].

The fruit fly, *Drosophila melanogaster*, is an emerging model system for sleep research [20], [21]. It has a simple central brain system that has streamlined the identification of novel sleep regulators and the mechanisms of sleep regulation [22]. The potassium channel, *Shaker*, was the first novel sleep-related gene found in *Drosophila* [23], and cAMP and protein kinase A (PKA) have been shown to regulate sleep in this model organism [24]. The mushroom body (MB) neurons, lateral ventral neurons (LNvs), pars intercerebralis (PI) neurons and dorsal fan-shaped body (FB) neurons constitute the brain regions known to be involved in regulating sleep in *Drosophila* [25]–[31], while the sleep-regulatory function of several monoamines, including dopamine, serotonin and octopamine, have also been elucidated [32]–[35].

In *Drosophila*, histamine acts as a neurotransmitter for photoreception and temperature sensing [36]–[40], and pharmacological tests have shown that it acts as a wake-activator [20]. Consistent with this function, administration of hydroxyzine, a histamine-receptor antagonist, was shown to increase sleep and reduce its latency [20]. However, the specific sleep/wake-controlling function of histamine and the *hisCl1* and *ort* genes, which encode histamine-gated chloride channels that act as histamine receptors [41], [42], have not yet been examined in detail.

Additionally, histamine is expressed in the eyelet axons and 18 cell bodies in protocerebrum which are designated HP1–4 and 2 cell bodies in the subesophageal ganglion (SOG) which are designated HS1 [40], [43]. The HP 3 neurons innervate the lobular and lateral protocerebrum in each hemisphere [43]. Even though the central body which containing mushroom body is devoid of histamine neuron fibers, histamine neurons are detected adjacent to the circadian clock neurons such as LNvs or dorsal neurons (DN) and HisCl1 receptor was identified in large LNvs [40], [43], [44]. According to these previous studies, we supposed that histaminergic signaling may be involved in sleep and circadian behavior in *Drosophila*.

Here, we used hypomorphic mutants and sleep profiles to reveal that the histamine-synthesizing HDC enzyme and the HisCl1 receptor have wake-promoting function, whereas the Ort receptor does not appear to have any sleep/wake regulatory function. Pharmacological and genetic approaches confirmed the wake-activating function of HDC and HisCl1-mediated histamine signaling. Furthermore, we identified PDF neurons as the source of the wake-activating function of the HisCl1 receptor pathway. Thus, although both HisCl1 and Ort play critical roles in photoreception and temperature sensing [39], [40], only the HisCl1 pathway appears to play a wake-promoting role. This study is the first to report the sleep/wake regulatory function of histamine receptors in *Drosophila*. Understanding the histaminergic wake-activating system in *Drosophila* can provide helpful clues for human sleep research.

## Results

### Defects in the *hdc* Gene Cause Extended Sleep

**Figure 1:**
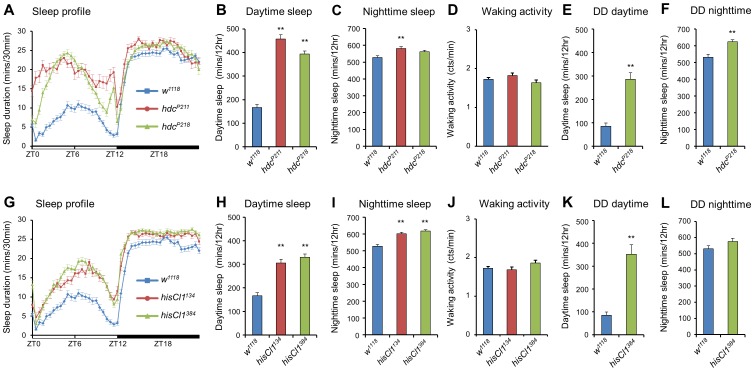
Loss-of function mutants of *hdc* or *hisCl1* have increased sleep durations. (**A**) Sleep profiles of *hdc* mutants in 12 hr:12 hr light dark (L:D). (**B, C**) The *hdc* mutants, *hdc^P211^* (n = 24) and *hdc^P218^* (n = 45), have longer sleep durations compared to control flies (*w^1118^*, n = 68). (**D**) The *hdc^P211^* and *hdc^P218^* mutants have waking activity levels similar to that of the control (*w^1118^*). (**E, F**) The daytime and nighttime sleep duration of *hdc^P218^* (n = 20) flies is increased compared to wild-type (n = 27) in constant darkness (DD). (**G**) Sleep profiles of *hisCl1* mutants in 12 hr:12 hr light dark (L:D). (**H, I**) The *hisCl1* mutants, *hisCl1^134^* (n = 50) and *hisCl1^384^* (n = 57), have longer sleep durations compared to control flies. (**J**) The *hisCl1^134^* and *hisCl1^384^* have waking activity levels similar to that of control flies (*w^1118^*). (**K**) The daytime sleep durations of *hisCl1^384^* (n = 17) are increased compared to that of wild-type flies (*w^1118^*, n = 27) in constant darkness (DD). (**L**) The nighttime sleep duration of *hisCl1^384^* (n = 17) flies is longer than that of wild-type (n = 27) in constant darkness (DD), but it is not significant enough. All flies were 4∼6-day-old females. The results were averaged over two days. Data are represented as mean ± s.e.m. (**, p<0.01; Student’s *t* test).

To test the function of histamine in the regulation of sleep and wakefulness in *Drosophila*, we tested loss-of function histamine signaling mutants. First, we tested two hypomorphic mutants of the *hdc* gene, *hdc^P211^* and *hdc^P218^*, which express lower levels of the *hdc* gene compared to wild-type flies (*w^1118^*) [39]. Daytime sleep durations of the outcrossed *hdc* mutants were significantly longer than those of wild-type flies (Figure 1A, B). The daytime sleep-length extension was approximately 200 min in *hdc* mutants, and nighttime sleep durations were also increased but to a lesser degree than daytime (Figure 1C). The waking activity of the mutants was similar to that of wild-type flies (Figure 1D), suggesting that the longer sleep in *hdc* mutants was not due to their inactivity, but rather due to an actual increase in their sleep duration. The number and average duration of daytime sleep episodes in *hdc^P211^* and *hdc^P218^* increased compared to wild-type flies. However, those of nighttime sleep episodes were not changed (Figure S1A–D). This may be due to a ceiling effect, since wild type files sleep for most of the nighttime. These results show that histamine acts as a wake-promoting factor and has a critical role in maintenance of the waking state.

Previous studies demonstrated that histamine and its receptors are involved in photoreception of *Drosophila*, and histamine signaling mutants have been found to exhibit visual system defects [39]. To exclude the possibility that a defect in the visual system contributed to the changes in sleep patterns observed in the *hdc* mutants, we evaluated the sleep phenotypes of *hdc* mutants in constant darkness. We reasoned that if defects in the visual system affected sleep in *hdc* mutants, then altered sleep phenotypes should not be found in constant darkness. However, the *hdc* mutant flies also showed increased sleep duration in constant darkness (Figure 1E, F). These results show that defects in the visual system do not cause sleep extensions observed in the *hdc* mutants.

Moreover, trans-heterozygotic mutants of *hdc^P211^* and *hdc^P218^* showed increased sleep durations similar to those of the homozygous *hdc^P211^* and *hdc^P218^* mutants (Figure S2A, B), whereas heterozygotes of *hdc^P211^* and *hdc^P218^* had a sleep duration similar to controls. These results suggest that neither the genetic background nor additional mutations contributed to the alteration of sleep patterns in the *hdc* mutants. Although these data were obtained from female flies, male *hdc* mutants also had longer sleep durations compared to wild-type flies (Figure S3A).

### The HisCl1 Receptor is Involved in Sleep-regulatory Mechanisms

To understand the role of histamine signaling in sleep regulation, we investigated the sleep-regulatory function of two histamine receptors. First, we tested the sleep phenotypes of *hisCl1* deletion mutants, *hisCl1^134^* (containing a 1.7-kbp deletion) and *hisCl1^384^* (containing a 1.0-kbp deletion from the 5′-end) [40]. These *hisCl1* mutant flies showed elevated sleep durations (Figure 1G). Increase of both daytime and nighttime sleep was significant, but increase of the daytime sleep was greater than increase in nighttime sleep. The daytime sleep durations were increased by 150 min in *hisCl1* deletion mutants compared to wild-type flies (*w^1118^*) (Figure 1H, I). The waking activities of the mutants were unchanged (Figure 1J), indicating that our results reflected an increased sleep duration rather than inactivity. The number and average duration of daytime sleep episodes were increased in *hisCl1* mutant flies (Figure S1E, G), further suggesting that the downstream signaling of the HisCl1 receptor is involved in sleep initiation and maintenance. However, the number and average duration of nighttime sleep episodes did not show a significant change (Figure S1F, H). As we mentioned previously, this would be ceiling effect of nighttime sleep.

Since HisCl1 also functions in light perception [37], we assessed sleep phenotypes of *hisCl1* mutants in constant darkness to exclude the possibility that defects in the visual system affected their sleep patterns. The *hisCl1* mutant flies showed increased sleep durations compared to wild-type flies in constant darkness (Figure 1K, L). This result means that defects in the visual system do not cause the sleep extensions observed in the *hisCl1* mutants.

To exclude the possible genetic background effects, we evaluated sleep durations in heterozygotes and trans-heterozygotes of the *hisCl1* mutants. The sleep durations of heterozygotes of the *hisCl1* mutants were similar to those of wild-type flies, but were longer in the trans-heterozygotes of *hisCl1^134^* and *hisCl1^384^* (Figure S2C, D), indicating that the genetic background did not affect the experimental outcome. Additionally, the male flies showed similar sleep pattern to those of female flies (Figure S3B). Collectively, these findings indicate that the histamine receptor, HisCl1, can activate wakefulness and that the activation pathway may be shared with the HDC enzyme. Thus, our results suggest that the histamine-HisCl1 receptor axis can activate and maintain wakefulness in *Drosophila*.

### The Ort Receptor is not Involved in Sleep-regulatory Mechanisms

**Figure 2:**
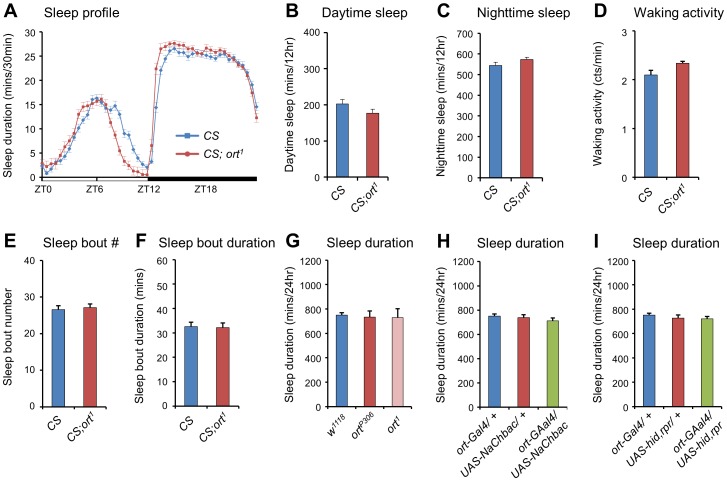
Loss-of-function mutants of the *ort* gene have sleep phenotypes similar to those of control flies. (**A**) Sleep profiles of the *ort* mutant in 12 hr:12 hr light dark (L:D). *CS* (*Canton-S*, n = 57) is the isogenic background of the mutant. (**B, C**) The *ort* mutant, *CS;ort^1^* (*Canton-S;ort^1^*, n = 68) has sleep durations similar to those of control flies (*CS*). (**D–F**) *CS;ort^1^* has a waking activity level, sleep-bout number and sleep-bout duration similar to those of control flies (*CS*). (**G**) The *ort* mutants, *ort^P306^* (n = 17) and *ort^1^* (n = 12), have sleep durations similar to those of control flies (*w^1118^,* n = 20). (**H, I**) The sleep duration of *ort* neuron-activated flies (*ort-Gal4/UAS-NaChBac*, n = 28) and *ort* neuron-ablated flies (o*rt-Gal4*/*UAS-hid,rpr*, n = 43) are unchanged compared to those of control flies (n = 32∼45). All flies were 4∼6-day-old females. The results were averaged over two days. Data are represented as mean ± s.e.m. (Student’s *t* test, two-sided Student’s *t* test).

To further explore the sleep-regulatory effects of histamine receptors, we tested the role of the Ort receptor using the mutant *CS;ort^1^*, which has a 569-bp deletion encompassing the second and third exons and is unable to produce a complete form of the Ort receptor [45]. Daytime and nighttime sleep durations of the *ort* mutant were similar to those of its isogenic control (*Canton-S*) (Figure 2A–C), demonstrating that histamine signaling through Ort does not participate in the wake-promoting pathway in *Drosophila.* The other sleep parameters of the *ort* mutant were also the same as those of wild-type flies (Figure 2D–F). To exclude the possible effects of genetic background, we outcrossed the *CS;ort^1^* mutant to the white-eyed background (*w^1118^*), and found out that the sleep duration of the white-eyed *ort^1^* mutant was similar to that of the isogenic background. In addition, the sleep duration in another *ort* mutant *ort^P306^* was also similar to that of the isogenic background (Figure 2G) [42]. These results mean that the genetic background did not affect sleep phenotypes of the *ort* mutants. Moreover, either activation or inactivation of *ort*-expressing neurons via *ort-Gal4* did not alter sleep patterns (Figure 2H, I), which means that the genetic manipulation of the Ort receptor did not affect sleep. Additionally, sleep durations of male *ort* mutants were similar to those of wild type males (Figure S3C), indicating that the wake activating histamine signaling is not gender biased. Therefore, although the HisCl1 and Ort receptors are both involved in light- and temperature-sensing mechanisms [39], [40], our results indicate that only the HisCl1 receptor is involved in the sleep/wake regulatory pathways of *Drosophila*.

### The PDF Neurons are Essential for the Wake Activation of Histamine Signaling

**Figure 3:**
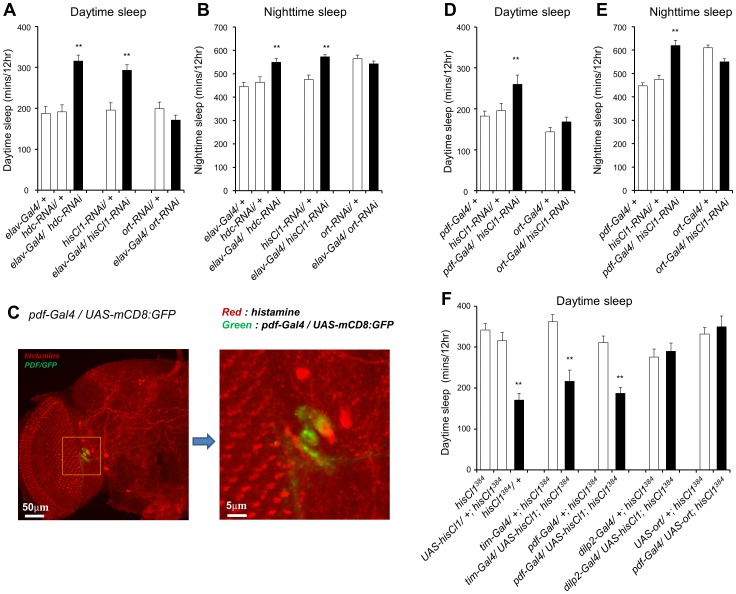
The PDF neurons are essential for the wake activation of histamine receptor signaling. (**A, B**) The expression of either *hdc-RNAi* or *hisCl1-RNAi* via *elav-Gal4* (*elav-Gal4/hdc-RNAi*, n = 30, *elav-Gal4/hisCl1-RNAi*, n = 54) increases the daytime and nighttime sleep duration compared to each control (n = 27∼59). However, the expression of *ort-RNAi* via *elav-Gal4* (*elav-Gal4/ort-RNAi*, n = 28) cannot increase the sleep duration compared to each control (n = 27∼59). (**C**) Anti-histamine staining partially co-localizes with GFP-expressing PDF neurons in *pdf-Gal4/UAS-mCD8:GFP* flies. (**D, E**) The expression of *hisCl1-RNAi* using *pdf-Gal4* (*pdf-Gal4/hisCl1-RNAi*, n = 23) increases the daytime and nighttime sleep duration compared to each control (n = 25∼56). However, the expression of *hisCl-RNAi* via *ort-Gal4* (*ort-Gal4/hisCl1-RNAi*, n = 28) cannot increase the sleep duration compared to each control (n = 39∼56) (**F**) The expression of *UAS-hisCl1* using either *tim-Gal4* or *pdf-Gal4* restores the increased sleep duration of *hisCl1^384^*, whereas ectopic expression of *UAS-hisCl1 via dilp2-Gal4* or *UAS-ort* via *pdf-Gal4* does not (n = 19∼43). All flies were 4∼6-day-old females. The results were averaged over two days. Data are represented as mean ± s.e.m. (**, p<0.01; two-sided Student’s *t* test).

To confirm the wake-activating function of the histamine-HisCl1 receptor axis, we suppressed the expression of histamine signaling genes using RNAi lines. Knockdown of *hdc* or *hisCl1* by targeted expression of either *hdc-RNAi* or *hisCl1-RNAi* using pan-neuronal *elav-Gal4* significantly increased sleep duration, whereas the knockdown of Ort receptor using *ort-RNAi* did not (Figure 3A, B). Reverse transcription-polymerase chain reaction (RT-PCR) demonstrated that transcript level in RNAi expressing flies was indeed lower than those of heterozygotic controls (Figure S4A–C). These results reconfirm that HDC and HisCl1 receptors have wake-promoting function, but the Ort receptor does not.

Histamine is mainly expressed in 10 cell bodies in each hemisphere of the *Drosophila* brain [43]. Moreover, our anti-histamine staining reconfirmed a previous study showing histamine-expressing cells being located near PDF neurons (Figure 3C) [44]. HisCl1 receptor was reported to be expressed in l-LNvs [40], suggesting that PDF neurons can receive histaminergic wake-activation signals, which could arise via the secretion of histamine from the HP2 or HP3 cell bodies in each hemisphere [43]. To clarify that the HisCl1 receptor in PDF neurons has a wake-promoting function, we reduced *hisCl1* gene expression in PDF neurons using *hisCl1-RNAi* via *pdf-Gal4*. As predicted, the daytime and nighttime sleep duration was increased in *hisCl1*-knockdown flies (Figure 3D, E). However, knockdown of *hisCl1* by targeted expression of *hisCl1-RNAi* in *ort*-expressing neurons using *ort-Gal4* did not extend the sleep duration (Figure 3D, E).

Next, we performed genetic restoration experiments in which we restored the expression of *hisCl1* gene in the mutant background using a *UAS-hisCl1* line. To verify the overexpression of *hisCl1* gene via the Gal4/UAS system, the transcript level of *hisCl1* was monitored by reverse transcription-polymerase chain reaction (RT-PCR) (Figure S4D). The expression of *UAS-hisCl1* using either *tim-Gal4* or *pdf-Gal4* in *hisCl1* mutant flies restored the increased daytime sleep duration back to the wild-type level. In contrast, expression of either *UAS-hisCl1* via *dilp2-Gal4* or *UAS-ort via pdf-Gal4* did not restore the increased daytime sleep duration of *hisCl1* mutant flies (Figure 3F). Together, these results strongly support that histamine has a wake-promoting function, specifically via the HisCl1 receptor in PDF neurons.

### Sleep Duration in *hdc* Mutants is Reduced by Histamine Treatment

**Figure 4:**
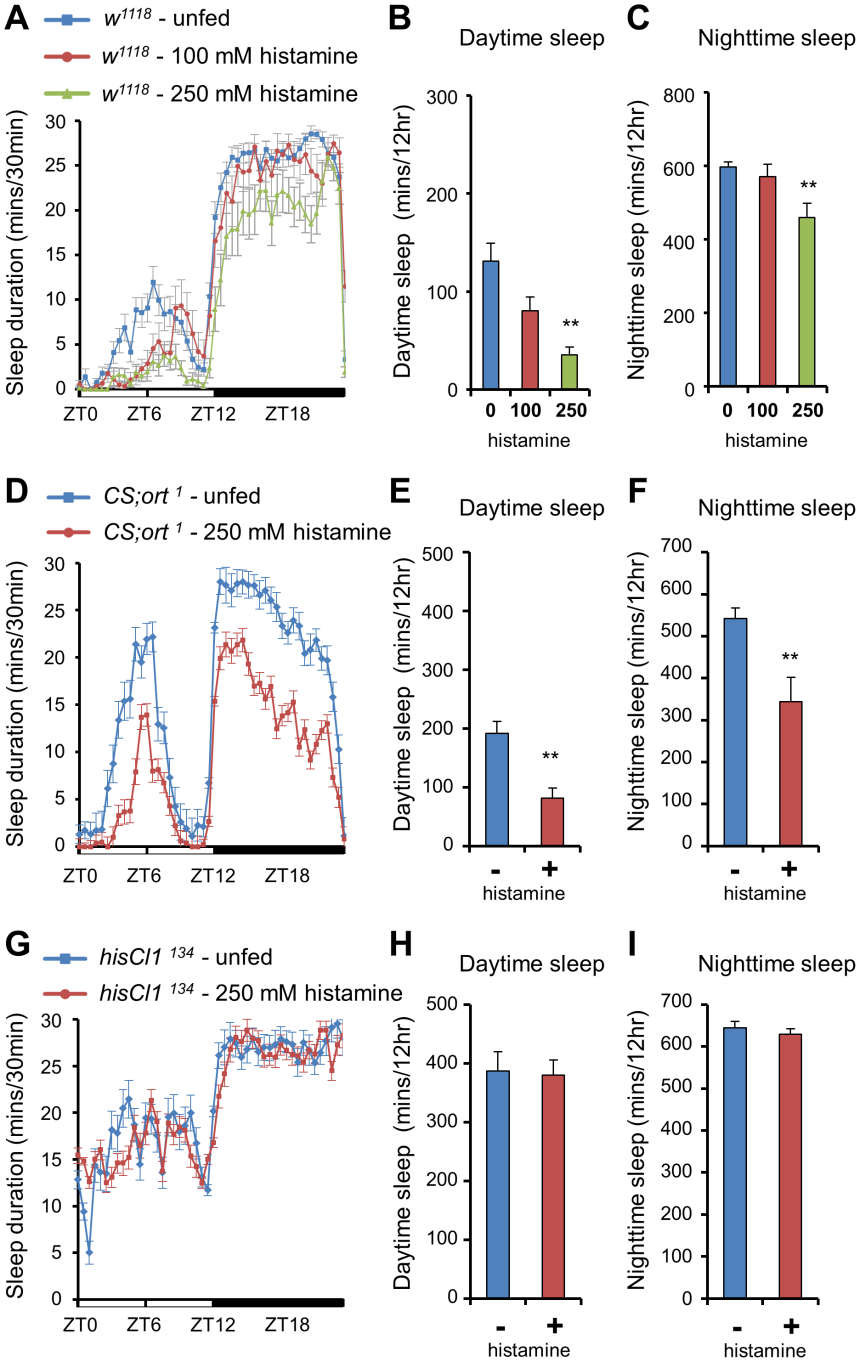
Histamine treatment dose-dependently decreases sleep duration and it decreases sleep duration of *ort* mutant but not of *hisCl*1 mutants. **(A–C)** Wild-type flies (*w^1118^*) were exposed to food containing 100 mM (n = 14) and 250 mM (n = 21) histamine, or to histamine untreated food (n = 28). The sleep duration is dose-dependently reduced in histamine-fed flies compared to unfed flies. **(D–F)** *CS;ort^1^* flies fed with 250 mM histamine (n = 15) show a reduced sleep duration compared to unfed controls (*CS;ort^1^*, n = 25), during both daytime and nighttime. **(G–I)** Histamine administration does not reduce the sleep duration of *hisCl1^134^* flies (n = 27) compared to unfed controls (n = 27). All flies were 4∼6-day-old females. Data are represented as mean ± s.e.m. (**, p<0.01; Student’s *t* test).

To activate histamine signaling through pharmacological means, we administered the exogenous histamine to wild type flies. To determine the optimal level of exogenous histamine, we fed wild-type flies 100 mM and 250 mM of histamine and examined their sleep behaviors. When flies were fed 100 mM of histamine, the daytime sleep duration was decreased but was not significant enough. In contrast, treating with 250 mM of histamine caused significant daytime and nighttime sleep reductions (Figure 4A–C), but did not cause any difference in food preference between histamine containing and non-containing food (data not shown). Moreover, administration of 250 mM histamine decreased the sleep duration in *ort* mutants but not in *hisCl1* mutants (Figure 4D–I). Additionally, administration of 250 mM of histamine could not shorten the sleep duration in flies with a pan-neuronal knockdown of *hisCl1* (Figure S5), indicating that histamine administration could activate the waking state only through the HisCl1 receptor.

**Figure 5:**
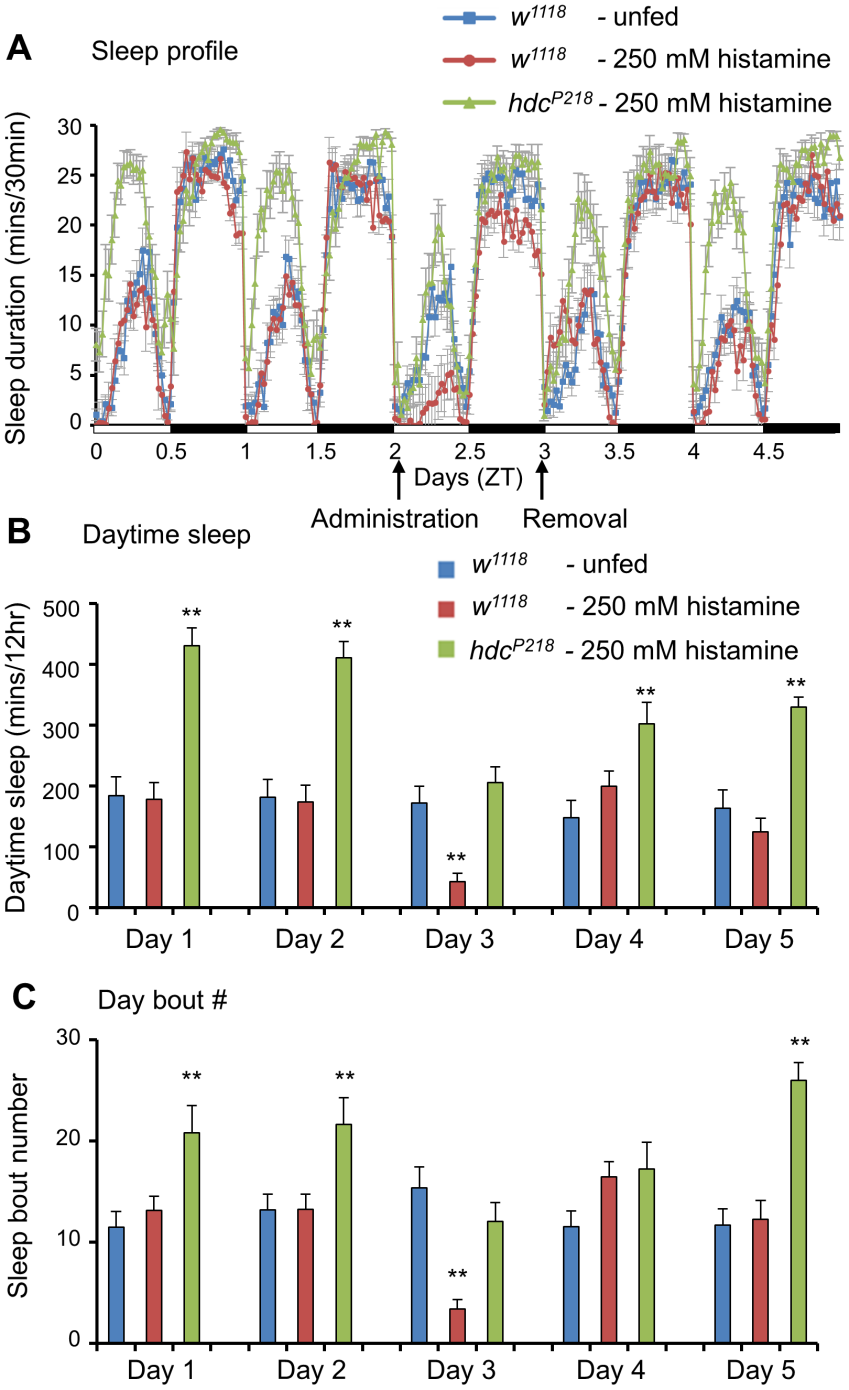
The administration of histamine decreases the sleep duration in control flies and *hdc* mutants. (**A, B**) Sleep profiles and daytime sleep durations of histamine-fed and -unfed flies. Days 1 and 2 show baseline recordings prior to histamine treatment. At 9∶00 a.m. of day 3, 250 mM histamine was fed to wild-type (*w^1118^*, n = 19) and *hdc^P218^* flies (*hdc^P218^*, n = 18), while a control group (*w^1118^*, n = 18) was not fed histamine. At 9∶00 a.m. of day 4, all flies were transferred to fresh histamine-untreated food. (**C**) Daytime sleep-bout numbers of histamine-fed and -unfed flies. All flies were 4∼6-day-old females. Data are represented as mean ± s.e.m. (**, p<0.01; Student’s *t* test).

When 250 mM histamine was fed on day 3, sleep duration was reduced by 150–200 min and this sleep reduction occurred during both daytime and nighttime (Figure 5A, B). On the first day of recovery (day 4), histamine-fed *w^1118^* flies showed sleep rebounds due to the prior day’s sleep loss. On the second day of recovery (day 5), the histamine-fed *w^1118^* flies showed a sleep pattern similar to that of the histamine-unfed *w^1118^* flies. Collectively, these findings suggest that histamine administration reversibly reduced sleep duration in wild-type flies.

Next, we tested whether histamine treatment could restore the increased sleep duration seen in *hdc^P218^* flies. During the baseline days (days 1 and 2), *hdc^P218^* flies showed increased sleep durations compared to *w^1118^* flies. After the *hdc^P218^* flies were fed with 250 mM histamine (day 3), their sleep pattern changed and resembled the sleep pattern of histamine-unfed *w^1118^* flies (Figure 5A, B). By day 5 (second day of recovery), the sleep duration was once again extended in histamine-fed *hdc^P218^* flies. These results suggest that histamine administration causes *hdc^P218^* flies to exhibit a wild-type sleep pattern.

As expected, histamine administration reduced the number of sleep episodes in the tested lines. When histamine treatment activated histamine signaling in *hdc^P218^* flies, the increase in the number of daytime sleep episodes was restored back to the wild-type level (Figure 5C). After 2 days of recovery from histamine administration, the number of daytime sleep episodes in *hdc^P218^* flies increased back to the level seen in *hdc^P218^* flies before histamine administration. By day 4 (first day of recovery), the number of daytime sleep episodes of histamine-fed *w^1118^* flies surpassed the number of daytime sleep episodes of histamine-unfed *w^1118^*, indicating that the recovery of sleep-bout number was faster than the recovery of sleep duration. Taken together, these data show that histamine administration activates and maintains the wake-promoting signal which does not cause any irreversible defects in the neuronal system of *Drosophila*.

### Histamine Administration Reconfirms that the PDF Neurons are Important for Wake-activation by Histamine Signaling

**Figure 6:**
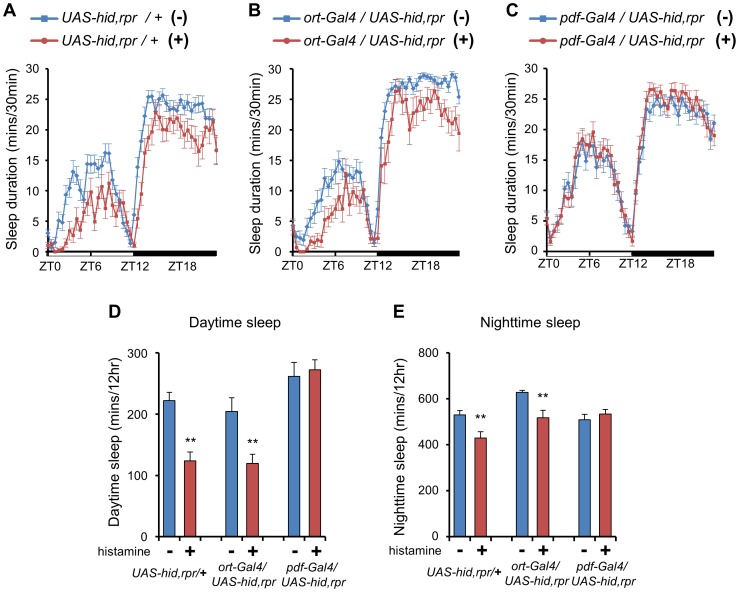
The administration of histamine does not decrease the sleep duration in PDF neuron-ablated flies. (**A, D, E**) Histamine-fed heterozygotes of the *UAS-hid,rpr* line *(UAS-hid,rpr/+*, n = 23) show a reduced sleep duration compared to unfed heterozygous controls (n = 32). (**B, D, E**) Histamine administration reduces sleep duration of *ort* neuron-ablated flies (n = 15) compared to untreated *ort* neuron-ablated flies (n = 27). (**C, D, E**) Following histamine administration, *pdf* neuron-ablated flies (n = 36) show a sleep duration similar to that of untreated *pdf* neuron-ablated flies (n = 34). (+) and (–) indicate the 250 mM histamine-fed and histamine-unfed flies respectively. All flies were 4∼6-day-old females. Data are represented as mean ± s.e.m. (**, p<0.01; Student’s *t* test).

To further identify the brain regions that modulate wake-activation by histamine signaling, we fed histamine to *UAS-hid,rpr* expressing flies; these flies express *reaper* and *hid*, which can ablate specific brain regions when placed under the control of different Gal4 drivers [46], [47]. We reasoned that if the ablated brain regions are important in wake-activation by histamine, then histamine treatment should not change the sleep durations when specific brain regions were ablated. If they are not involved, however, histamine administration should produce a sleep-reduction phenotype similar to that seen in wild-type flies. As a control, histamine was administrated to heterozygotes of the *UAS-hid,rpr* line (*UAS-hid,rpr*/+). Histamine-fed *UAS-hid,rpr* heterozygotes showed a reduction in sleep duration comparable to that of histamine-unfed *UAS-hid,rpr* heterozygotes during both daytime and nighttime (Figure 6A, D, E), suggesting that the genetic background did not affect the wake-activating function of histamine administration in these flies. Next, 250 mM histamine was fed to *ort* neuron-ablated flies. We found out that the sleep duration of the histamine-fed line was significantly decreased during both daytime and nighttime, providing a pharmacological confirmation that *ort* neurons (and thus Ort receptors) are not important in wake-activation by histamine signaling (Figure 6B, D, E).

The circadian rhythms of both *hdc* and *hisCl1* mutants were normal in constant darkness (Table S1). These results suggest that PDF neurons may regulate sleep and circadian rhythms using independent signaling pathways. Moreover, The expression of PDF was unchanged in either *hdc* or *hisCl1* mutants and administration of 250 mM histamine decreased the sleep duration in the loss-of-function mutant of the *pdf* gene (*pdf^01^*) (data not shown). Thus, we reasoned that if PDF neurons are important in wake activation, then histamine administration should not reduce sleep duration in the *pdf-Gal4/UAS-hid,rpr* line, in which the ablation of PDF neurons was confirmed by PDF staining (data not shown). Our results revealed that administration of 250 mM histamine to PDF neuron-ablated flies could not reduce their daytime and nighttime sleep duration, confirming that PDF neurons are important for wake-activation by histamine (Figure 6C–E).

Because using *UAS-hid,rpr* to ablate cells can cause several defects during developmental stages, we used an *UAS-shibire^ts^* line in which the targeted Gal4 expressing synapses are conditionally blocked only at a high temperature [48]–[50]. Using the *UAS-shibire^ts^* line along with *pdf-Gal4,* we showed that conditional synaptic blocking in PDF neurons inhibited the sleep reduction caused by 250 mM administration of histamine (Figure S6). This further confirms that PDF neurons can regulate histaminergic wake-promoting signals.

## Discussion

Using genetic and pharmacological methods to manipulate histamine signaling, we show that the HisCl1 receptor and its downstream signaling cascade regulate wake-evoking behavior in *Drosophila*, while Ort receptor does not show any sleep/wake regulatory function. Histamine promotes activity via the HisCl1 receptor. Reduced histamine in HDC mutants or loss of the HisCl1 receptor both show reduced activity and enhanced sleep. Additionally, the relevant signaling pathway downstream of the HisCl1 receptor may function in the PDF neurons. Finally, we demonstrate that the histamine-HisCl1 receptor axis can activate and maintain wakefulness in PDF neurons.

These data show the complete functional segregation of the two histamine receptors for the first time. Ort receptor is expressed in large monopolar cells (LMC), postsynaptic to photoreceptors in the lamina and is a major target of photoreceptor synaptic transmission in *Drosophila*. In contrast to Ort, HisCl1 receptor is not expressed in postsynaptic neurons of photoreceptors. It is expressed in lamina glia and shapes the LMC postsynaptic response of Ort signaling [37]. Both Ort and HisCl1 receptor are involved in temperature-preference behaviors [40], but the major independent function of HisCl1 receptor remains elusive. In this study, we found out that sleep regulation is a novel and independent function of HisCl1 receptor. Additionally, this finding is an important clue in understanding the functional evolution of the two histamine receptors in *Drosophila*.

We propose that wake-activation by histamine signaling in *Drosophila* is similar to that found in mammals. We found out that *hdc* mutant flies have increased sleep durations compared to controls and a previous study showed that HDC-knockout mice have increased paradoxical sleep compared to controls [10]. This suggests that the HDC enzyme has a common wake-promoting function in mammals and flies. However, the structures of histamine receptors differ between flies and mammals; the histamine receptors of *Drosophila* are histamine-gated chloride channels, whereas the mammalian histamine receptors belong to the rhodopsin-like G-protein-coupled receptor family [11], [45]. Currently, researchers are working to identify a metabotropic histamine receptor in *Drosophila* [43], [51]. Despite the structural differences of the mammalian and *Drosophila* receptors, they share a wake-activating function. This functional homology may be the result of evolution and gives us a hint to find out the metabotropic histamine receptors in *Drosophila*.

Surprisingly, functional conservations between flies and mammals are also found among the histamine receptor subtypes. The HisCl1 receptor has a wake-activating role, whereas the Ort receptor does not. This result parallels differences in the wake-activation roles of the H_1_ and H_2_ receptors in mammals: the H_1_ receptor can activate wakefulness, but the H_2_ receptor cannot [14], [15]. Thus, our data provide a more detailed understanding of the potential functional relationship between the HisCl1 and H_1_ receptors. A functional connection between the Ort receptor and the H_2_ receptor is also possible, since the two have little effect on sleep/wake regulation in their corresponding model systems. No auto-receptor of histamine has yet been found in *Drosophila*, suggesting that there may not be a *Drosophila* homolog for the mammalian H_3_ receptor. Further research should shed greater light on the evolutionary relationship between the histamine receptors of flies and mammals.

Histamine signaling modulates the maintenance of wakefulness and controls light sensing, and we speculate that a number of interactions are possible between these two different pathways. Previous studies on light-perception mechanisms showed that histamine mutants exhibit light-sensing defects [36]–[39]. However, we found out that the sleep duration was increased in histamine signaling mutants compared to wild-type flies in constant darkness (Figure 1F and 2F). Thus, the perception of light in the context of evoking wakefulness is independent of vision-related light perception in *Drosophila*. Further research will be required to definitively establish the relationship between light perception and sleep regulation.

Previous studies revealed that the PDF neurons promote wakefulness in *Drosophila* [28], [52]. Our findings show that histamine signaling acts as a wake-promoting pathway in PDF neurons. The HisCl1 receptor is a chloride channel, which would be expected to inhibit the function of the neurons. However, since previous studies showed that chloride channels can activate the function of the neurons [53], [54], hence the HisCl1 receptor might be an activator of the PDF neurons. The downstream signaling of histamine-HisCl1 receptor in PDF neurons should be further studied using genetic manipulation and electro-physiological methods.

Orexin is a neuropeptide that acts as an important wake-activating neurotransmitter in mammals, as shown by the demonstration that defects in orexin synthesis can cause narcoleptic symptoms in human and animals [6], [55]–[57]. Orexin neurons activate wakefulness in the lateral hypothalamic area and the feedback loop between orexin neurons and monoaminergic neurons such as histaminergic and serotonergic neurons (tuberomammillary nucleus, TMN, and dorsal raphe nucleus, DR) controls wakefulness in the hypothalamus and the brain stem [6], [7], [55], [58]–[60]. Histamine receptors are essential for wake-activation by orexin treatment [61], indicating that orexin and histamine signaling constitute an interactive wake-activating system in mammals. However, orexin has not been found in *Drosophila*. A previous study suggested that the PDF neuropeptide functions similar to those of orexin in *Drosophila* [52], potentially explaining many aspects of the wake-activation cascade in *Drosophila*. Histamine and orexin have similar wake-activating function, but mammalian histamine mutants do not show narcoleptic symptoms. Here, we show that histamine and one of its receptors, HisCl1, constitute an important wake-evoking axis in *Drosophila*. Moreover, we demonstrate that histamine-signaling mutants cannot maintain wakefulness during the daytime, which is similar to the phenotype of orexin mutants in mammals. Hence, we propose that, in *Drosophila*, histamine may have a function similar to that of the mammalian orexin. Further research is required to establish the functional relationship between wake activation of histamine signaling in *Drosophila* and wake-promoting function of orexin and histaminergic system in mammals.

## Materials and Methods

### Fly Strains

All flies were reared on standard cornmeal-yeast-agar medium at 25°C under 12 h light/12 h dark conditions (LD cycle). The *hisCl1^134^*, *hisCl1^384^*, *ort-Gal4*, and isogenic control *w^1118^* flies were graciously provided by Dr. Hong (KAIST, Republic of Korea, Daejeon) [40]. The *pdf-Gal4* [62], *tim-Gal4* [63], *dilp2-Gal4* [64], *UAS-shibire^ts^* [48]–[50] lines were as described previously. The *hdc^P211^*, *hdc^P218^* and *ort^P306^* flies were kindly provided by Dr. W. Pak (Purdue University, West Lafayette, IN) [39]. The *UAS-hid,rpr* flies were provided by Paul Taghert (Washington University, St. Louis, MO). The *elav-Gal4* flies were provided by Young-Joon Kim (GIST, Republic of Korea, Gwangju). The *Canton-S* (1), *CS;ort^1^* (1133), *UAS-mCD8:GFP* (5137) and *UAS-NaChBac* (9466) flies were obtained from the Bloomington *Drosophila* Stock Center (Bloomington, IN). The *hdc-RNAi* (transformant ID : 34621, construct ID : 10972), *hisCl1-RNAi* (transformant ID : 104966, construct ID : 112578) and *ort-RNAi* (transformant ID : 107363, construct ID : 106461) lines were obtained from the Vienna *Drosophila* RNAi Center (VDRC, Vienna, Austria). *Canton-S* is the isogenic background for *CS;ort^1^*. To exclude the possibility that the altered sleep pattern was caused by the genetic background, we outcrossed *hdc* and *hisCl1 mutants* to *w^1118^*. Before genetic manipulation, all of the Gal4 and UAS lines were outcrossed to *w^1118^*. To construct *UAS-hisCl1*, ∼1.7 kb of the *hisCl1* cDNA was cloned into a UAS vector modified for integration using the φC31 system [65], and then the transgene was inserted was into a specific second chromosome site (defined here as VIE-72a) [66].

### Sleep Analysis

To examine sleep behavior, 4∼6-day-old female flies were placed in 65 mm×5 mm glass vials containing 4% sucrose and 2% agar, and sleep behavior was monitored using *Drosophila* Activity Monitors (DAMs) (Trikinetics, Waltham, MA) in a chamber kept under constant temperature (25°C) and humidity (60%). Locomotor activity data were collected under LD (12 h light/12 h dark) or DD (constant darkness) [67]. We gathered beam-crossing numbers of DAMs every 1 min. Sleep was defined as over 5 min of inactivity [68], and a custom-developed sleep analysis program was used to obtain sleep parameters (total sleep time, waking activity, sleep-bout number and sleep-bout duration). For statistical analysis, student's t-tests and one-way ANOVA tests were conducted using Microsoft Excel (Microsoft).

### Conditional Neuronal Inhibition using *UAS-shibire^ts^*

To inhibit PDF neurons conditionally, we ectopically expressed *UAS-shibire^ts^*, which expresses vesicle recycling dynamin at synapse. The specific form of thermo-sensitive dynamin causes rapid neurotransmitter blocking at temperature over 29°C, but not under 21°C. We reared the flies under 21°C and loaded the flies to DAM monitor on day 3 after the eclosion. After that, we recorded the baseline sleep behavior at 21°C before the neuronal inhibition. On day 5, we recorded the sleep patterns at 29°C and compared the sleep patterns between *shibire^ts^* expressing and unexpressing flies.

### RT-PCR

Total RNA was extracted using TRIzol (Invitrogen), reverse transcription was performed using M-MLV reverse transcriptase (Promega), and RT-PCR was performed using specific primers (for *hdc*, 5′- CGC GAT CCT CAC CAG TCA ACC -3′ and 5′- AGA GCA GCA GTG GTG TCA CCA A -3′; for *hisCl1*, 5′- CTC AAC AGG TAA CTT CAC CTG C -3′ and 5′- ACG GGA AGA AAA AGC GCG AGA A -3′; for *ort*, 5′- TGC TCC TCC TGG GGG CAG CAA -3′ and 5′- AGA GAG CTA GCG AAA GTA TTT AC -3′; for *rp49* as a control, 5′- ATC CGC CCA GCA TAC AG -3′ and 5′- TCC GAC CAG GTT ACA AGA A -3′). The reaction mixture was initially denatured for 5 min at 94°C and then subjected to 30 cycles at 94°C for 30 s, 55°C for 30 s, 72°C for 1 min and a final 72°C extension for 7 min.

### Behavior Assays Following Pharmacological Treatments

Histamine diphosphate (Sigma, St. Louis, MO; catalog number, 53310) was dissolved in distilled water (DW) and mixed with 4% sucrose and 2% agar (100 mM and 250 mM). We recorded two days of baseline sleep before drug administration, and then moved flies from screening vials (4% sucrose and 2% agar) to the drug-treated vials (4% sucrose, 2% agar and histamine diphosphate) at 9∶00 a.m. After one day of drug administration, the flies in the drug-treated vials were returned to normal screening vials, and behavioral recording was continued for two more days to monitor the sleep recovery of histamine-fed flies.

### Immunohistochemistry (IHC)

For histamine antibody staining, tissues were fixed in 4% 1-ethyl-3-(3-dimethylaminopropyl) carbodiimide hydrochloride (Sigma) dissolved in PBST, blocked with 3% normal goat serum in PBST, and then incubated overnight with anti-histamine (1∶500 in blocking solution; Immunostar, Inc., Hudson, WI) at 4°C. The tissues were then washed with PBST and incubated with rhodamine-conjugated goat anti-rabbit (1∶250; Jackson ImmunoResearch). Tissues were mounted in a VECTASHIELD Mounting Medium (Vector Laboratories, Burlingame, CA) and examined by confocal microscopy (LSM510; Zeiss, Thornwood, NY).

## Supporting Information

Figure S1 **Sleep parameters of histamine-signaling mutant flies. (A, C)** The daytime sleep-bout number and average duration of *hdc^P211^* (n = 24) and *hdc^P218^* (n = 45) flies are elevated compared to control flies (*w^1118^*, n = 68). **(B, D)** The nighttime sleep-bout number and durations of the *hdc^P211^* and *hdc^P218^* mutants are similar to those of the control (*w^1118^*). **(E, G)** The daytime sleep-bout numbers and average duration of *hisCl1^134^* and *hisCl1^384^* are elevated compared to control flies (*w^1118^*). **(F, H)** The nighttime sleep-bout number and durations of the *hisCl1^134^* (n = 50) and *hisCl1^384^* (n = 57) mutants are similar to those of the control (*w^1118^*). All flies were 4∼6-day-old females. The results were averaged over two days. Data are represented as mean ± s.e.m. (**, p<0.01; Student’s *t* test).
(TIF)

Figure S2 **Trans-heterozygotes of either** ***hdc*** **or** ***hisCl1*** **mutants have increased sleep durations. (A, B)** The heterozygous *hdc^P218^* mutants, *hdc^P211^/+* (n = 16) and *hdc^P218^/+* (n = 16), have sleep durations similar to that of their wild-type control, *w^1118^* (*+/+*, n = 68). The trans-heterozygous mutant of *hdc^P211^* and *hdc^P218^* (*hdc^P218^/hdc^P211^*, n = 38) shows an increased sleep duration similar to that of the homozygous *hdc* mutants. **(C, D)** The heterozygous *hisCl1* mutants, *hisCl1^134^/+* (n = 14) and *hisCl1^384^/+* (n = 15), have sleep durations similar to that of their wild-type control, *w^1118^* (*+/+*, n = 68), but the trans-heterozygote of *hisCl1^134^* and *hisCl1^384^* (*hisCl1^134^/hisCl1^384^*, n = 35) shows a longer sleep duration. All flies were 4∼6-day-old females. The results were averaged over two days. Data are represented as mean ± s.e.m. (**, p<0.01; one-way ANOVA).
(TIF)

Figure S3 **Sleep patterns of male histamine-signaling mutant flies. (A)** Sleep profiles of *hdc* male mutants in 12 hr:12 hr light dark (L:D). Male flies of the *hdc* mutant lines, *hdc^P211^* (n = 29) and *hdc^P218^* (n = 29), have increased daytime sleep durations compared to control flies (*w^1118^*, n = 49). **(B)** Sleep profiles of *hisCl1* male mutants in 12 hr:12 hr light dark (L:D). The male flies of the *hisCl1* mutant lines, *hisCl1^134^* (n = 38) and *hisCl1^384^* (n = 50), have increased daytime sleep durations compared to control flies (*w^1118^*, n = 49). **(C)** Sleep profiles of *hdc* male mutants in 12 hr:12 hr light dark (L:D). Male flies of the *ort* mutant line, *CS;ort^1^* (n = 23), have sleep patterns similar to those of control flies (*Canton-S*, n = 46). All flies were 4∼6-day-old females. The results were averaged over two days. Data are represented as mean ± s.e.m. (**, p<0.01; Student’s *t* test).
(TIF)

Figure S4 **Transcripts of** ***hdc*** **,** ***hisCl1*** **and** ***ort*** **gene in RNAi expressing flies were lower than those of heterozygotic controls. (A–C)** RT-PCR results showing that the *hdc-RNAi, hisCl1-RNAi* and *ort-RNAi* lines suppress the expression of *hdc, hisCl1* and *ort* gene via pan-neuronal *elav-Gal4.* **(D)** The *UAS-hisCl1* line overexpresses *hisCl1* gene via *elav-Gal4.*
(TIF)

Figure S5 **The administration of histamine does not reduce sleep duration in** ***hisCl1*** **knockdown mutants. (A–C)** Sleep profiles and sleep durations of histamine-fed and -unfed *elav-Gal4/+ and elav-Gal4/hdc-RNAi* flies. Daytime and nighttime sleep durations are reduced in histamine-fed *elav-Gal4/+* (n = 13) and *elav-Gal4/hdc-RNAi* (n = 28) flies compared to histamine-unfed *elav-Gal4/+* (n = 26) and *elav-Gal4/hdc-RNAi* (n = 32) flies. **(D–F)** Sleep profiles and sleep durations of histamine-fed and -unfed *ort-RNAi/+ and elav-Gal4/ort-RNAi* flies. Daytime and nighttime sleep durations are reduced in histamine-fed *ort-RNAi/+* (n = 20) and *elav-Gal4/ort-RNAi* (n = 18) flies compared to those in histamine-unfed *ort-RNAi/+* (n = 18) and *elav-Gal4/ort-RNAi* (n = 40) flies. **(G–I)** Sleep profiles and sleep durations of histamine-fed and -unfed *hisCl1-RNAi/+ and elav-Gal4/hisCl1-RNAi* flies. Daytime and nighttime sleep durations are reduced in histamine-fed *hisCl1-RNAi/+* (n = 14) flies compared to those in histamine-unfed *hisCl1-RNAi/+* (n = 15) flies. However, histamine-fed *elav-Gal4/hisCl1-RNAi* (n = 28) line shows similar sleep durations compared to histamine-unfed *elav-Gal4/hisCl1-RNAi* (n = 49), during both daytime and nighttime. (+) and (–) indicate the 250 mM histamine-fed and -unfed flies respectively. All flies were 4∼6-day-old females. Data are represented as mean ± s.e.m. (**, p<0.01; Student’s *t* test).
(TIF)

Figure S6 **The administration of histamine does not decrease the sleep durations in PDF neuron-inhibited flies. (A, C, D)** Histamine-fed *pdf-Gal4/+* (n = 25) flies show a reduced sleep duration compared to the untreated control (*pdf-Gal4/+,* n = 27) at 29°C, during both daytime and nighttime. **(B, C, D)** Histamine administration does not decrease the sleep duration of *pdf* neuron-inhibited flies (*pdf-Gal4/UAS-shibire^ts^*, n = 61) compared to untreated controls (*pdf-Gal4/UAS-shibire^ts^*, n = 36) at 29°C, during both daytime and nighttime. All flies were 4∼6-day-old females. Data are represented as mean ± s.e.m. (**, p<0.01; Student’s *t* test).
(TIF)

Table S1 **Histamine signaling mutants show normal locomotor activity rhythms in constant darkness.**
(TIF)
