# Supplementary figures and images for: Histamine-HisCl1 Receptor Axis Regulates Wake-Promoting Signals in Drosophila melanogaster
Source: PLoS One. 2013 Jul 3;8(7):e68269. doi: 10.1371/journal.pone.0068269 (PMC3700972; doi:10.1371/journal.pone.0068269)

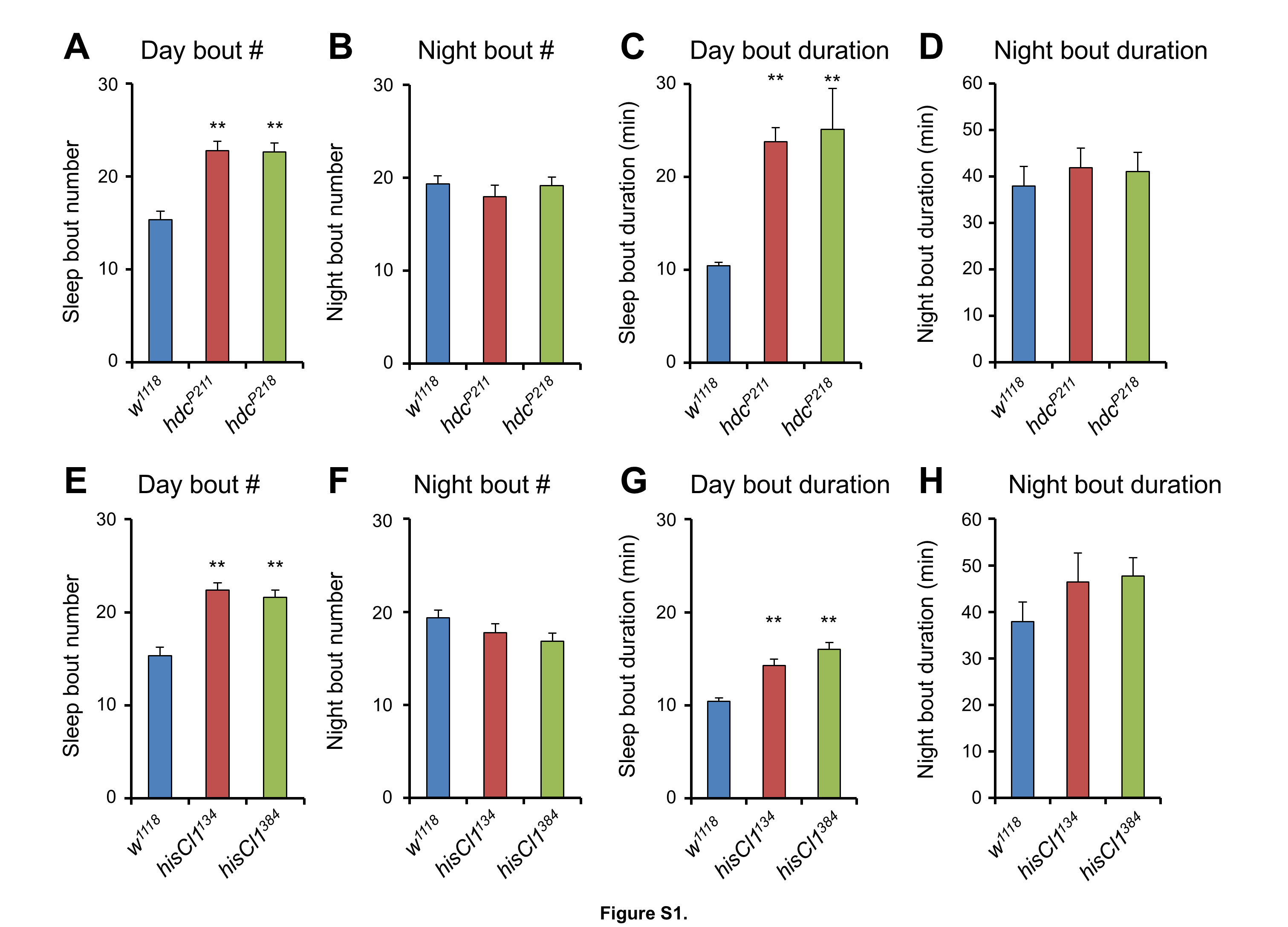

Supplement: Figure S1 — Sleep parameters of histamine-signaling mutant flies. (A, C) The daytime sleep-bout number and average duration of hdcP211 (n = 24) and hdcP218 (n = 45) flies are elevated compared to control flies (w1118, n = 68). (B, D) The nighttime sleep-bout number and durations of the hdcP211 and hdcP218 mutants are similar to those of the control (w1118). (E, G) The daytime sleep-bout numbers and average duration of hisCl1134 and hisCl1384 are elevated compared to control flies (w1118). (F, H) The nighttime sleep-bout number and durations of the hisCl1134 (n = 50) and hisCl1384 (n = 57) mutants are similar to those of the control (w1118). All flies were 4∼6-day-old females. The results were averaged over two days. Data are represented as mean ± s.e.m. (**, p<0.01; Student’s t test). (TIF) [file pone.0068269.s001.tif]

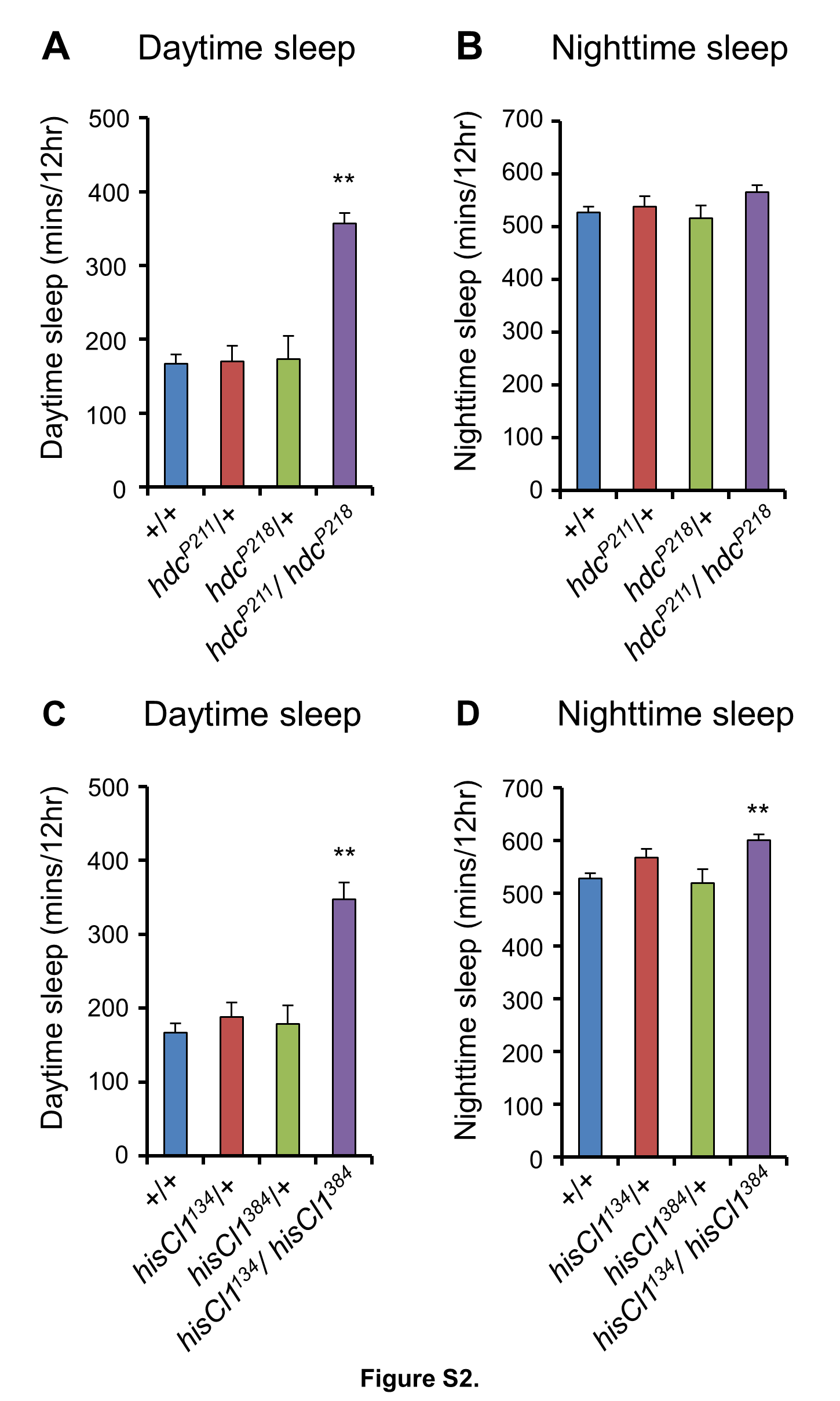

Supplement: Figure S2 — Trans-heterozygotes of either hdc or hisCl1 mutants have increased sleep durations. (A, B) The heterozygous hdcP218 mutants, hdcP211/+ (n = 16) and hdcP218/+ (n = 16), have sleep durations similar to that of their wild-type control, w1118 (+/+, n = 68). The trans-heterozygous mutant of hdcP211 and hdcP218 (hdcP218/hdcP211, n = 38) shows an increased sleep duration similar to that of the homozygous hdc mutants. (C, D) The heterozygous hisCl1 mutants, hisCl1134/+ (n = 14) and hisCl1384/+ (n = 15), have sleep durations similar to that of their wild-type control, w1118 (+/+, n = 68), but the trans-heterozygote of hisCl1134 and hisCl1384 (hisCl1134/hisCl1384, n = 35) shows a longer sleep duration. All flies were 4∼6-day-old females. The results were averaged over two days. Data are represented as mean ± s.e.m. (**, p<0.01; one-way ANOVA). (TIF) [file pone.0068269.s002.tif]

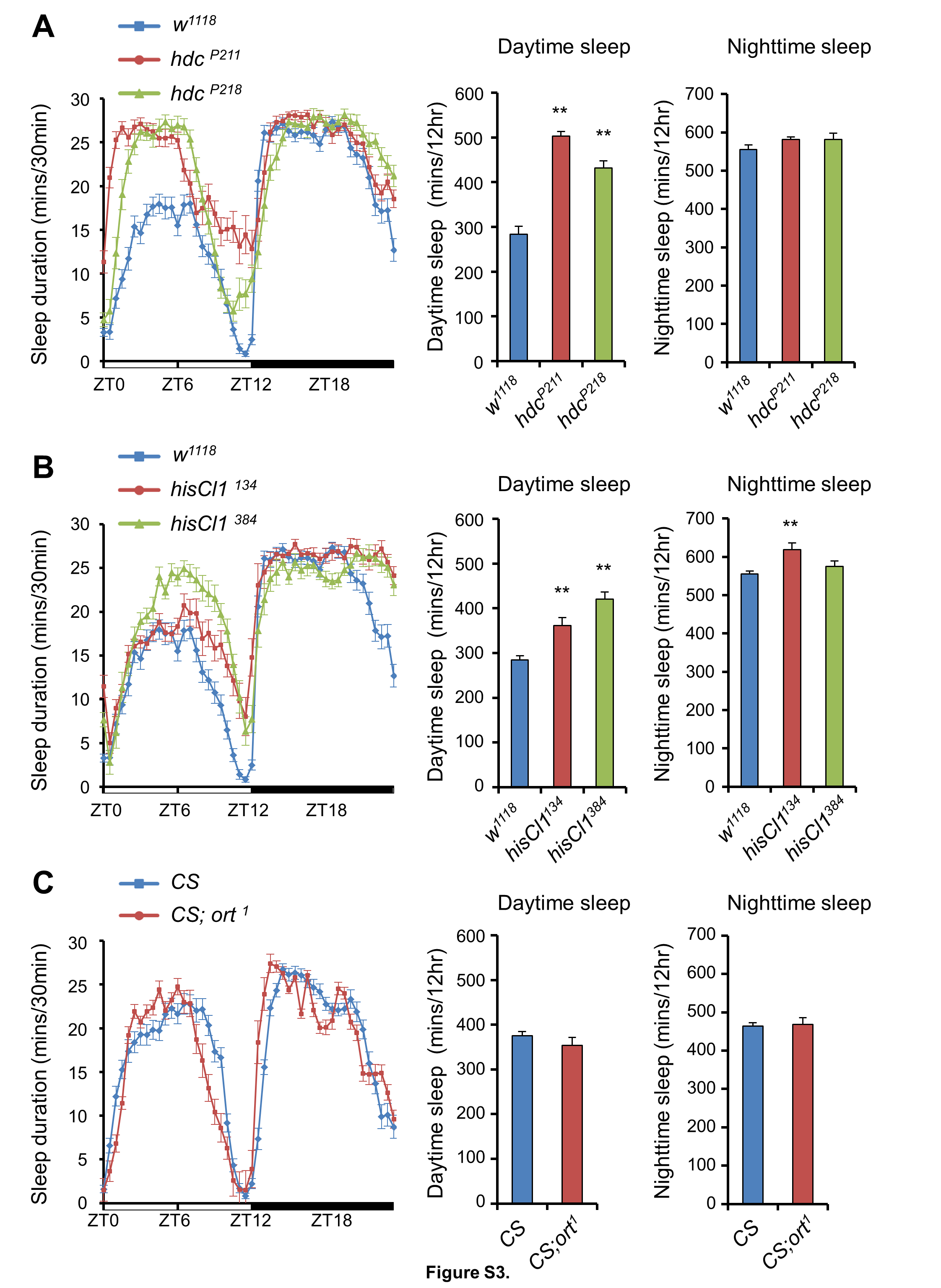

Supplement: Figure S3 — Sleep patterns of male histamine-signaling mutant flies. (A) Sleep profiles of hdc male mutants in 12 hr:12 hr light dark (L:D). Male flies of the hdc mutant lines, hdcP211 (n = 29) and hdcP218 (n = 29), have increased daytime sleep durations compared to control flies (w1118, n = 49). (B) Sleep profiles of hisCl1 male mutants in 12 hr:12 hr light dark (L:D). The male flies of the hisCl1 mutant lines, hisCl1134 (n = 38) and hisCl1384 (n = 50), have increased daytime sleep durations compared to control flies (w1118, n = 49). (C) Sleep profiles of hdc male mutants in 12 hr:12 hr light dark (L:D). Male flies of the ort mutant line, CS;ort1 (n = 23), have sleep patterns similar to those of control flies (Canton-S, n = 46). All flies were 4∼6-day-old females. The results were averaged over two days. Data are represented as mean ± s.e.m. (**, p<0.01; Student’s t test). (TIF) [file pone.0068269.s003.tif]

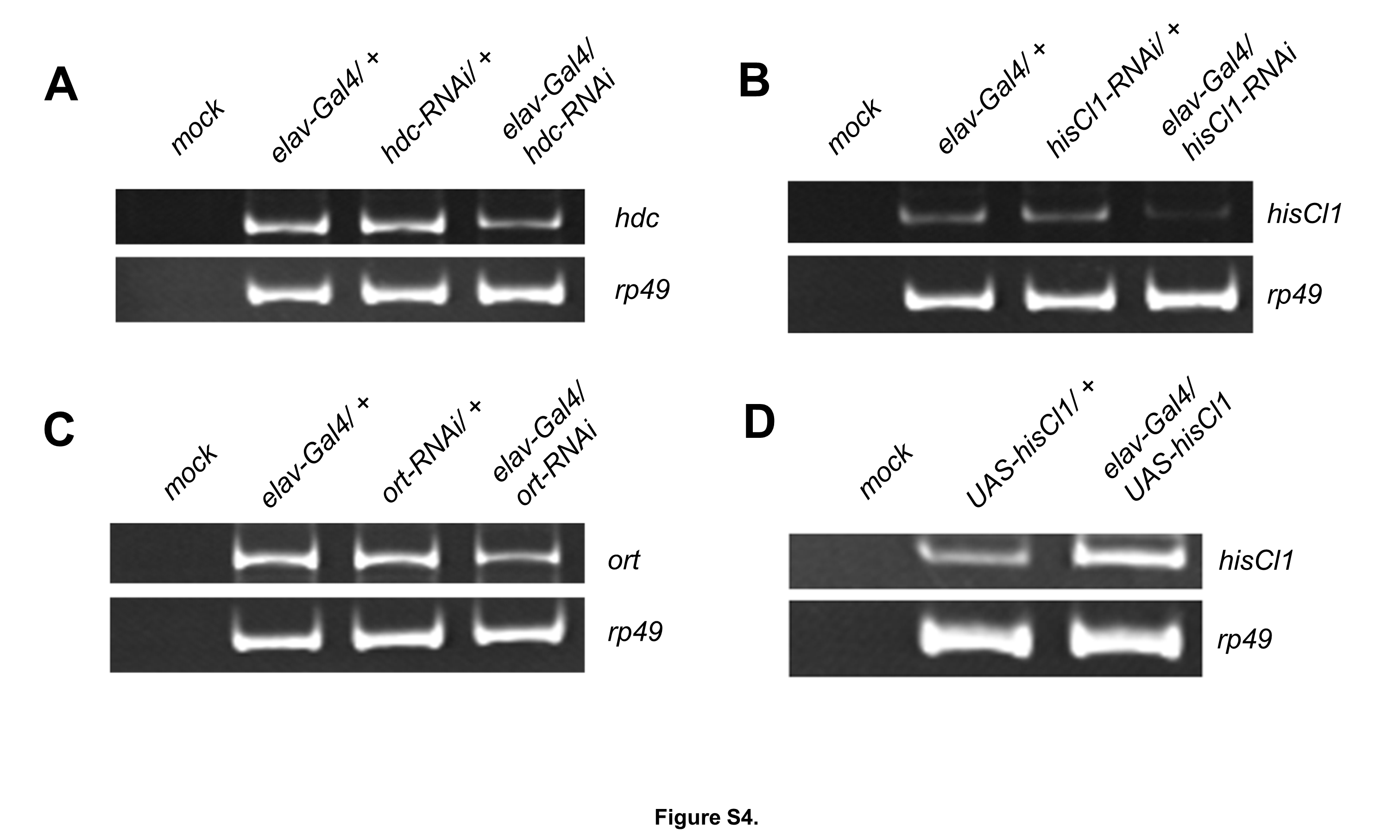

Supplement: Figure S4 — Transcripts of hdc , hisCl1 and ort gene in RNAi expressing flies were lower than those of heterozygotic controls. (A–C) RT-PCR results showing that the hdc-RNAi, hisCl1-RNAi and ort-RNAi lines suppress the expression of hdc, hisCl1 and ort gene via pan-neuronal elav-Gal4. (D) The UAS-hisCl1 line overexpresses hisCl1 gene via elav-Gal4. (TIF) [file pone.0068269.s004.tif]

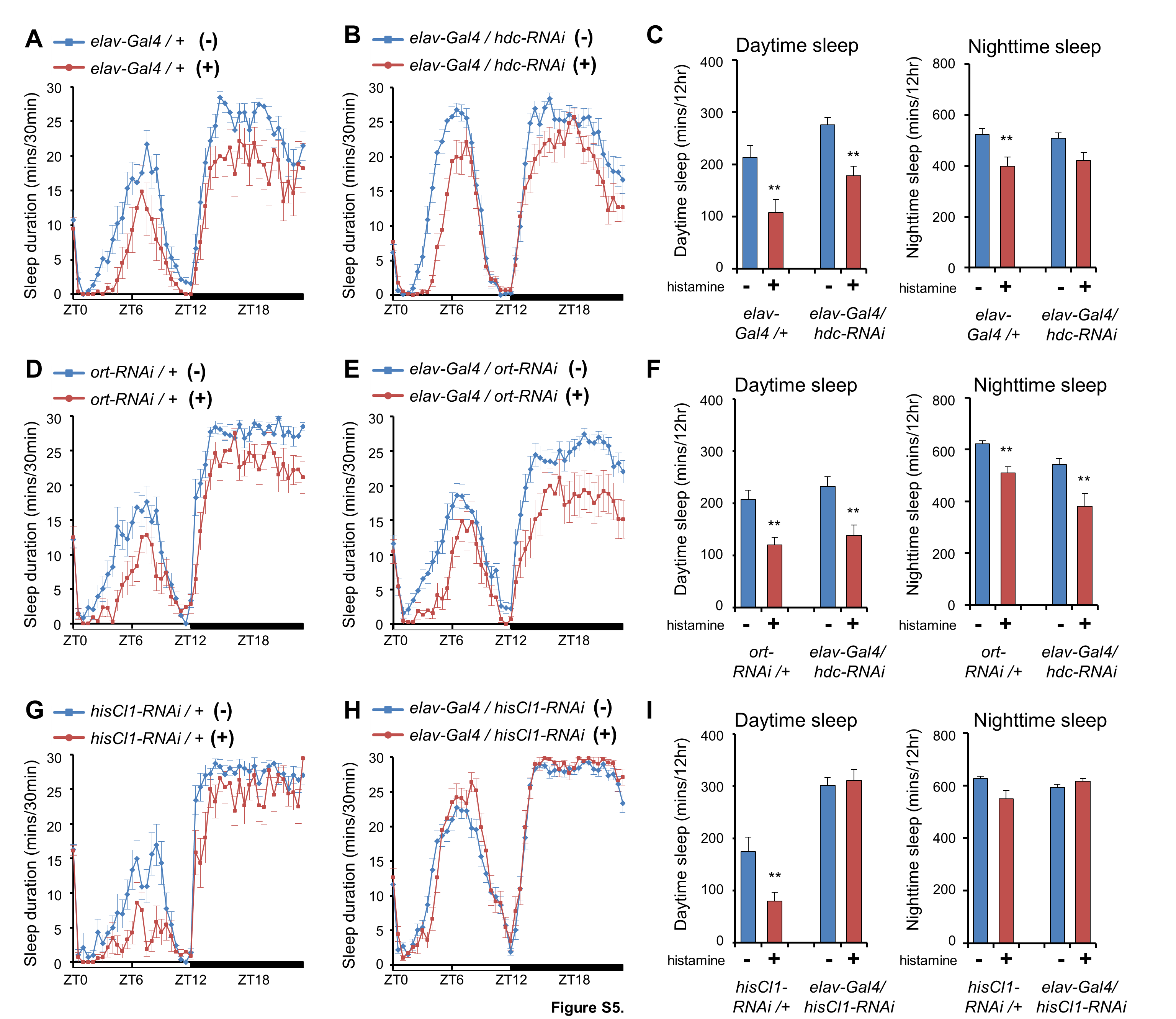

Supplement: Figure S5 — The administration of histamine does not reduce sleep duration in hisCl1 knockdown mutants. (A–C) Sleep profiles and sleep durations of histamine-fed and -unfed elav-Gal4/+ and elav-Gal4/hdc-RNAi flies. Daytime and nighttime sleep durations are reduced in histamine-fed elav-Gal4/+ (n = 13) and elav-Gal4/hdc-RNAi (n = 28) flies compared to histamine-unfed elav-Gal4/+ (n = 26) and elav-Gal4/hdc-RNAi (n = 32) flies. (D–F) Sleep profiles and sleep durations of histamine-fed and -unfed ort-RNAi/+ and elav-Gal4/ort-RNAi flies. Daytime and nighttime sleep durations are reduced in histamine-fed ort-RNAi/+ (n = 20) and elav-Gal4/ort-RNAi (n = 18) flies compared to those in histamine-unfed ort-RNAi/+ (n = 18) and elav-Gal4/ort-RNAi (n = 40) flies. (G–I) Sleep profiles and sleep durations of histamine-fed and -unfed hisCl1-RNAi/+ and elav-Gal4/hisCl1-RNAi flies. Daytime and nighttime sleep durations are reduced in histamine-fed hisCl1-RNAi/+ (n = 14) flies compared to those in histamine-unfed hisCl1-RNAi/+ (n = 15) flies. However, histamine-fed elav-Gal4/hisCl1-RNAi (n = 28) line shows similar sleep durations compared to histamine-unfed elav-Gal4/hisCl1-RNAi (n = 49), during both daytime and nighttime. (+) and (–) indicate the 250 mM histamine-fed and -unfed flies respectively. All flies were 4∼6-day-old females. Data are represented as mean ± s.e.m. (**, p<0.01; Student’s t test). (TIF) [file pone.0068269.s005.tif]

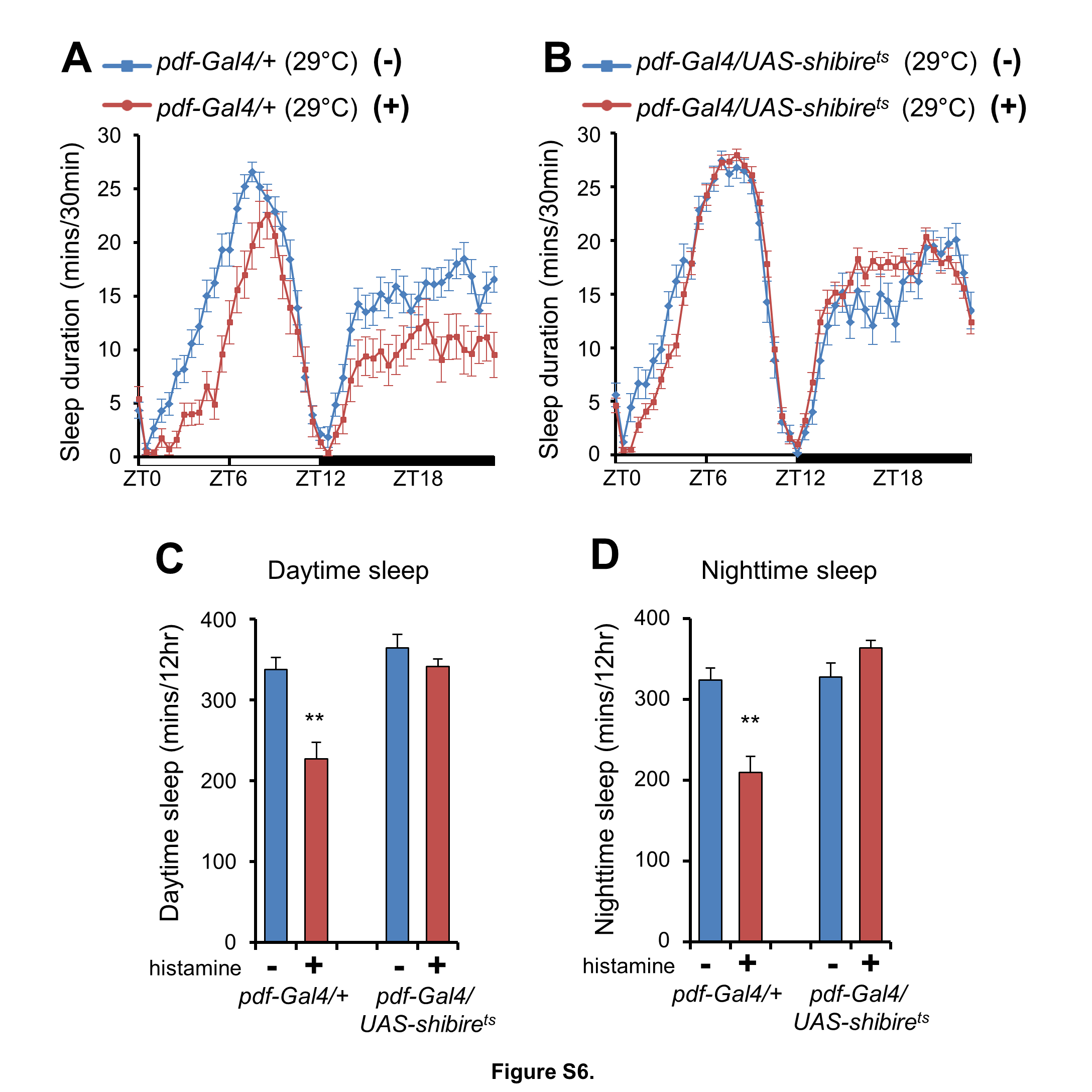

Supplement: Figure S6 — The administration of histamine does not decrease the sleep durations in PDF neuron-inhibited flies. (A, C, D) Histamine-fed pdf-Gal4/+ (n = 25) flies show a reduced sleep duration compared to the untreated control (pdf-Gal4/+, n = 27) at 29°C, during both daytime and nighttime. (B, C, D) Histamine administration does not decrease the sleep duration of pdf neuron-inhibited flies (pdf-Gal4/UAS-shibirets, n = 61) compared to untreated controls (pdf-Gal4/UAS-shibirets, n = 36) at 29°C, during both daytime and nighttime. All flies were 4∼6-day-old females. Data are represented as mean ± s.e.m. (**, p<0.01; Student’s t test). (TIF) [file pone.0068269.s006.tif]

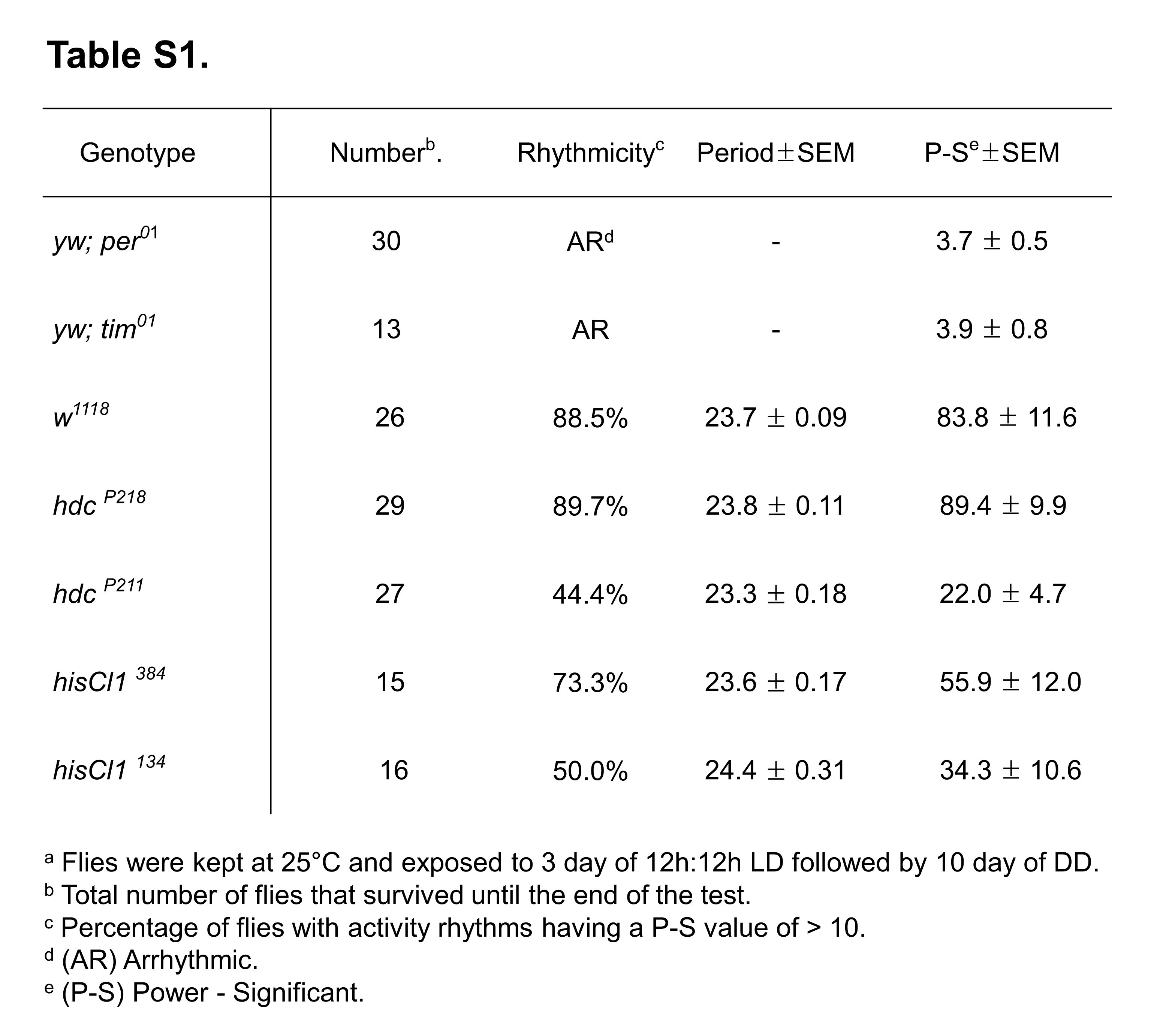

Supplement: Table S1 — Histamine signaling mutants show normal locomotor activity rhythms in constant darkness. (TIF) [file pone.0068269.s007.tif]
